# Genome-Wide Analysis Reveals Copy Number Variant Gene *TGFBR3* Regulates Pig Back Fat Deposition

**DOI:** 10.3390/ani14182657

**Published:** 2024-09-12

**Authors:** Chunlei Zhang, Huan Yang, Qinglei Xu, Mingzheng Liu, Xiaohuan Chao, Jiahao Chen, Bo Zhou

**Affiliations:** College of Animal Science and Technology, Nanjing Agricultural University, Nanjing 210095, China; 2020105039@stu.njau.edu.cn (C.Z.); 2021105038@stu.njau.edu.cn (H.Y.); 2019205004@njau.edu.cn (Q.X.); 2020205018@stu.njau.edu.cn (M.L.); 2021205020@stu.njau.edu.cn (X.C.); chenjiahao@stu.njau.edu.cn (J.C.)

**Keywords:** CNV, *TGFBR3*, Chinese indigenous pig, dose effect, eQTL

## Abstract

**Simple Summary:**

In this study, we conducted genome-wide copy number variation (CNV) analysis using next-generation sequencing data from Large White (LW) and Minzhu (MZ) pigs and integrated transcriptomic data from dorsal adipose tissues of 180-day-old LW and MZ pigs for expression quantitative trait loci (eQTL) analysis. Among the identified CNVs, the transforming growth factor beta receptor 3 (*TGFBR3*) gene was found to be associated with back fat thickness (BFT), with a dose effect observed between the *TGFBR3* gene at the genomic and transcriptomic levels. In vitro experiments further demonstrated that *TGFBR3* expression is involved in the proliferation and differentiation of porcine preadipocytes.

**Abstract:**

BFT is closely related to meat quality and lean meat percentage in pigs. The BFT traits of European LW pigs significantly differ from those of Chinese indigenous fatty MZ pigs. CNV is a prevalent genetic variation that plays an important role in economically important traits in pigs. However, the potential contribution of CNV to BFT in LW and MZ pigs remains unclear. In this study, whole-genome CNV detection was performed using next-generation sequencing data from LW and MZ pigs, and transcriptome data from back fat tissue of 180-day-old LW and MZ pigs were integrated for expression quantitative trait loci (eQTL) analysis. We identified a copy number variation in the *TGFBR3* gene associated with BFT, showing a dose effect between the genome and transcriptome levels of the *TGFBR3* gene. In porcine preadipocytes, *TGFBR3* expression continuously increased during differentiation. Knockdown of *TGFBR3* using specific siRNA inhibited preadipocyte differentiation and proliferation. Our study provides insights into the genetic regulation of pork quality and offers a theoretical basis for improving carcass quality by modulating BFT in pigs.

## 1. Introduction

Adipose tissue is a significant component of pig carcasses [1]. Over the past few decades, pig breeding programs have focused on increasing the lean meat percentage due to the growing demand for protein [2]. Back fat thickness (BFT) is closely related to the meat quality and lean meat percentage of pigs. BFT is primarily influenced by subcutaneous fat deposition, a dynamic process that involves fat synthesis, transport, and metabolism. Genes such as *FTO*, *METTL3*, and *IGF2BP1* have been reported to play crucial roles in fat deposition [3,4,5].

Copy number variation (CNV) is an important and common genetic variation characterized by DNA segments > 1 kb in length that vary in copy number relative to a reference genome [6]. CNVs encompass more base pairs than single nucleotide polymorphisms (SNPs) and have a greater impact on phenotypic variation, disease susceptibility, and species evolution [7,8]. Pigs, as economically important animals, have been found to harbor a large number of CNVs in their genomes. CNV was first reported in pigs by Fadista et al. (2008), who detected a total of 37 CNV regions (CNVRs) across the chromosomes of 12 Duroc pigs, laying the groundwork for understanding the association between CNV and economic traits [9]. For example, Meishan pigs, known for their larger litter size compared to Duroc pigs, exhibit a copy number duplication located in the *AHR* gene, which correlates with this trait [10]. Additionally, Chinese indigenous pigs display greater disease resistance than European pigs, consistent with the functions of genes annotated in CNVRs specific to Chinese pig breeds [11]. To date, CNV studies have been conducted in cattle [12], sheep [13], poultry [14], and mice [15].

Previous studies have also explored the relationship between CNV and BFT. In Italian Large White (LW) pigs, CNV was detected using the porcine SNP60 BeadChip, identifying a total of 170 CNVRs, with 16 significantly associated with BFT. Notably, *ZPLD1*, located within a significant CNVR, has also been linked to human obesity [16]. In Indian Landlly pigs, 267 CNVRs were detected on the autosomes, and the genes annotated in these CNVRs were implicated in back fat deposition [17]. However, most of these CNV studies have relied on SNP chip data, which are less comprehensive than resequencing data. Comparative studies on BFT between Chinese indigenous fatty pigs and European commercial pigs have not yet been conducted.

**Figure 1:**
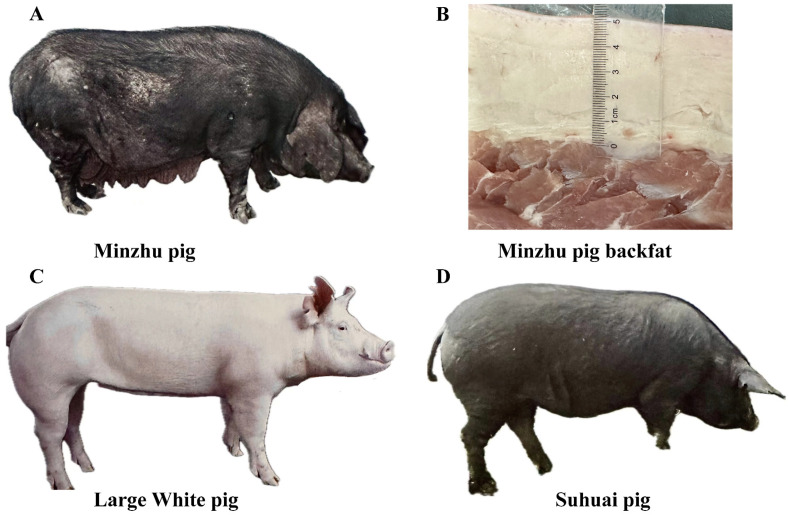
Pig breeds used in this study. (**A**) Minzhu pig (MZ). (**B**) Back fat of MZ at the last rib. (**C**) Large White (LW) pig. (**D**) Suhuai (SH) pig.

Compared to modern commercial lean pig breeds, most Chinese indigenous pig breeds have greater back fat deposits, resulting in lower carcass leanness and reduced economic efficiency. Examples of North China-type pig breeds are Minzhu (MZ) (Figure 1A,B) and Huai. Conversely, the LW pig (Figure 1C) is globally recognized as one of the most extensively bred commercial pig breeds, acclaimed for its growth performance, low BFT, and high carcass leanness [18,19]. However, the genetic determinants of CNV responsible for the significant disparity in BFT between MZ and LW pigs remain unidentified. This study aims to explore the potential effects of genomic CNV on the marked differences in BFT between LW and MZ pigs. To increase the credibility of our results, we selected Suhuai (SH) pigs (Figure 1D) as a reference population, which contains 75% LW and 25% Huai pigs. We detected genome-wide CNVRs in LW and MZ pigs and integrated transcriptomic data to identify CNVR-related candidate genes affecting BFT.

## 2. Materials and Methods

### 2.1. Pig Samples and Sequencing Data

The next-generation sequencing data of LW (*n* = 12) and MZ pigs (*n* = 11) were obtained from a public database (https://www.ncbi.nlm.nih.gov/, accessed on 2 August 2022) (Table S1). Twenty-three SH pigs were selected from the Huaiyin pig breeding farm, Huai’an, Jiangsu Province, China. The genomic DNA of SH pigs was extracted from ear tissue using a standard phenol/chloroform/isoamyl alcohol protocol, followed by whole-genome sequencing on the Illumina Hiseq2000 platform. FastQC (https://www.bioinformatics.babraham.ac.uk/projects/fastqc/, accessed on 2 July 2022) was used for quality control, and the cutadapt program (https://cutadapt.readthedocs.io/en/stable/, accessed on 10 July 2022) was used to filter and trim the reads. The MultiQC (v 1.11) was used to integrate the quality control results to meet CNV detection requirements [20]. The data were aligned to the reference genome (Sscrofa 11.1) using the Burrows–Wheeler Aligner (v 0.7.17) [21]. The overall average sequencing depth was 12.89×, with a highest of 16.22×, lowest of 9.17×, and average mapping ratio to the porcine reference genome of 96.47% for 46 samples (Table S1).

Transcriptomic data for LW (*n* = 3) and MZ pigs (*n* = 3) were obtained from the same public database (https://www.ncbi.nlm.nih.gov/, accessed on 2 August 2022) (Table S1). All pigs were aged 180 days, and their back fat tissue was sampled. Trimmomatic (v 0.39) was used to eliminate adapter sequences and low-quality reads to obtain clean reads [22]. The quality-controlled clean reads were used for downstream analysis. HISAT2 (v 2.0.5) was used to map all clean reads to the reference genome (Sscrofa 11.1), and the featureCounts program was used to count the number of reads across each sample. DESeq2 software (v 1.44.0) was used to identify differentially expressed genes (DEGs) between LW and MZ pigs [23,24,25]. A threshold of “log2 (fold change) > 2” and “adjusted *p*-value < 0.05” was used to assess the DEGs between LW and MZ pigs. The log2 (fold change) was calculated as log2 (FPKM_LW: FPKM_MZ). The default parameters were used for all data processing software mentioned above.

### 2.2. CNV Calling

CNV calling was analyzed using the CNVcaller software [26], which is based on the read depth strategy and contains fast generalized detection algorithms to detect CNVs in large-scale sequencing data with complex genomic structures. Here, a copy number below 1.5 was considered by the CNVcaller as copy number “loss”, between 1.5 and 2.5 as “normal”, and above 2.5 as copy number “gain”. The genome-wide CNVR map was drawn using RIdeogram (v 0.2.2) [27]. The Bedtools (v 2.29.0) program was used to convert the vCard File with CNVR information to browser extensible data (BED) format. ChipSeeker (v 1.20.0) annotated the CNVR position in the genome based on the BED file [28,29]. The g:Profiler was used to annotate genes in CNVRs [30]. Clustering analysis was performed in LW, MZ, and SH pigs using principal component analysis (PCA) in PLINK (v 1.90) [31].

### 2.3. Comparison between LW and MZ Pigs

The differential CNVRs between LW and MZ pigs were further investigated using TBTOOLS (v 1.098661) [32]. The DAVID database was used to perform gene ontology (GO) and Kyoto Encyclopedia of Genes and Genomes (KEGG) enrichment analyses [33]. The variance-stabilizing transformation (V_ST_) value of the shared but differential CNVRs between LW and MZ pigs was calculated by the formula [6]:

$$V_{ST}=\frac{V_{total}-\frac{V_{L}\times N_{L}+V_{M}\times N_{M}}{N_{total}}}{V_{total}}$$

where V_ST_ is based on an unbiased measure of the fixation index (F_ST_) [6]. V_total_ is the total variance calculated by the copy numbers. V_L_ and V_M_ are the variances calculated by copy numbers in LW and MZ pigs, respectively. N_L_ and N_M_ are the numbers of LW and MZ pigs, respectively. N_total_ is the total number of all pigs.

### 2.4. Screening of CNVR-Related Genes Associated with Fat Deposition by Multi-Omics Association Analysis

By a V_ST_ analysis, the CNVRs with the top 1% V_ST_ values were set to the preliminarily differential CNVRs. Subsequently, the IGV program (v 2.17.0) was used to screen the final differential CNVRs between LW and MZ pigs [34]. The R package ‘ggplot2’ (v 3.3.0) was used to draw a heat map and a volcano plot to show the global expression of multiple genes between LW and MZ pigs [35]. Matrix-eQTL (v 2.3) software was used to analyze expression quantitative trait loci (eQTL) to evaluate the relationship between CNVRs and gene expression changes [36]. The Cytoscape (v 3.7.1) program was used to draw an interactive network diagram revealing the regulatory relationship between CNVRs and genes [37].

### 2.5. QTL Analyses

Differential CNVRs with both trans-eQTL and cis-eQTL effects (*n* = 19) were selected and overlapped with the QTL data of the pigs (https://www.animalgenome.org/cgi-bin/QTLdb/SS/index, accessed on 1 October 2022). The number of QTLs overlapped by CNVRs was statistically analyzed based on the description of QTL traits.

### 2.6. Validation of CNV Type

Genomic DNA was extracted from the ear tissue samples using a standard protocol employing phenol/chloroform/isoamyl alcohol (MACKLIN, Shanghai, China). To validate the CNV type, four CNVRs were randomly selected and analyzed using quantitative polymerase chain reaction (qPCR) and the 2-ΔCt method (Vazyme, Nanjing, China) [38]. Specific primers were designed by the primer-BLAST tool (https://www.ncbi.nlm.nih.gov/tools/primer-blast, accessed on 2 August 2022), and the glucagon gene (*GCG*) with a highly conserved copy number was used as the internal reference gene [39]; the primers used for CNV type validation are listed in Table S3. All CNVRs were assessed on the QuantStudio 5 real-time PCR system (ABI, Foster, CA, USA), and PCR conditions were set according to the manufacturer’s instructions. A total volume of 20 µL was used for PCR and included the following components: 2 µL DNA (5 ng/μL), 10 µL SYBR master mix (2×), 0.4 µL of forward and reverse primers (20 pmol/μL), and 7.2 μL water. 

### 2.7. Cell Culture, Transfection, and Differentiation

The animals used in this study were 7-day-old Erhualian piglets. These piglets were from the Erhualian pig production cooperation (Changzhou, Jiangsu, China).

Subcutaneous adipose tissue was isolated from the back (above the thoracic spine) of euthanized piglets. The tissue was then immersed in phosphate-buffered saline (PBS) and digested with collagenase type I (Biosharp, Hefei, China) at 37 °C and 75 rpm/min on a shaker for 2 h. The digestion process was terminated by adding an equal volume of F12 medium (containing 10% fetal bovine serum (FBS) + 1% penicillin–streptomycin) (Gibco, CA, USA). The digested adipose tissue was filtered through a 100 μm nylon mesh, and the solution containing porcine preadipocytes was collected. Subsequently, the solution was centrifuged at 1000 rpm/min for 10 min to collect the preadipocytes. The porcine preadipocytes were cultured in a medium at 37 °C with a carbon dioxide concentration of 5%. The medium was changed every two days.

When the cell density reached 85% confluence, cells were cultured in 6-well or 12-well plates, and plasmids or oligonucleotides were transfected into the cells using LipoFiter 3.0 (Beyotime, Shanghai, China). The oligonucleotides used are listed in Supplementary Table S2 (GENEray, Shanghai, China).

Differentiation induction medium (DIM) was used to induce preadipocytes induced at a density of 85%. The DIM contains the following components: 2.5 µM dexamethasone, 8.6 µM insulin, 0.1 mM 3-isobutyl-1methylxanthine (IBMX) (MACKLIN, Shanghai, China), 1% penicillin–streptomycin, and 10% FBS in F12 medium. After 4 days of differentiation induction, the medium was changed to 10% FBS maintenance medium containing 8.6 µM insulin until day 8. The above medium was changed every two days. After the induction of preadipocytes differentiation was completed, the degree of preadipocytes differentiation was monitored by oil red staining.

### 2.8. RNA Isolation, cDNA Library Preparation, and qPCR

Total RNA was extracted from porcine subcutaneous adipocytes and reverse transcribed into standard cDNA and qPCR as per the experimental requirements (Vazyme, Nanjing, China). Porcine *GAPDH* was used as the internal reference gene to normalize the mRNA levels, and the primers used for qPCR amplification are listed in Table S3.

### 2.9. Oil Red O Staining and Triglyceride Assay

Differentiated adipocytes were washed twice with PBS and then fixed with 4% paraformaldehyde for 30 min (MACKLIN, Shanghai, China). Cells were washed twice with PBS after fixation, and the cells were then stained with 60% saturated oil red O for 30 min, followed by two washes with PBS (MACKLIN, Shanghai, China). Subsequently, the stained cells were observed using a Zeiss Axiovert 40 CFL inverted microscope (Zeiss, Jena, Germany). Total triglyceride content was quantified by eluting oil red O with isopropanol and measuring absorbance at 510 nm.

### 2.10. EdU Assay

Porcine preadipocytes were seeded in 12-well plates containing coverslips. Transfection commenced once the cell density reached 60%. After 24 h of transfection, the cells were incubated with 10 µM of EdU (Cy5) (APExBIO, Houston, TX, USA) for 4 h in a cell incubator. Removing the Cy5 solution, cells were fixed with paraformaldehyde for 30 min. After removal of paraformaldehyde, cells were rinsed with 3% BSA three times for 5 min each time, and cells were permeabilized with 0.3% Triton^®^ X-100 for 20 min after fixation. The staining reaction solution was configured according to the instructions to stain the cells for 30 min in a dark environment. After staining, Cy5 azide and Hoechst 33342 were imaged using a confocal microscope (Zeiss, Jena, Germany) with excitation wavelengths of 646 nm and 350 nm, respectively.

### 2.11. Statistical Analysis

The significance of cell experiments was analyzed using a *t*-test in IBM SPSS Statistics 26. Results are expressed as the mean ± SEM, and a *p*-value of less than 0.05 indicated a statistically significant difference.

## 3. Results

### 3.1. CNVR Detection in LW and MZ Pigs

**Figure 2:**
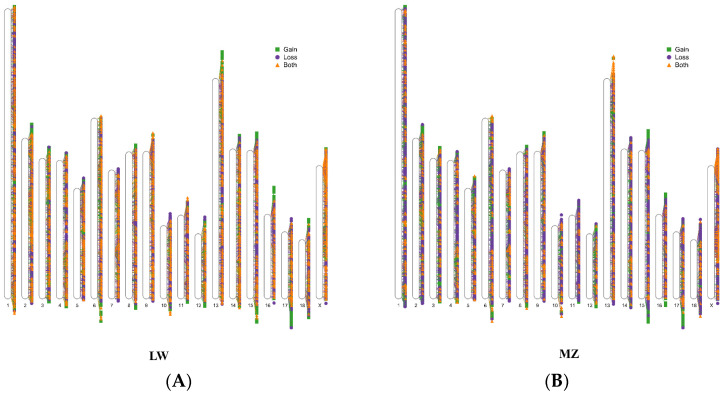
Whole-genome distribution of CNVRs and variation types in (**A**) LW and (**B**) MZ pigs. Green squares indicate the gain type, purple circles indicate the loss type, and yellow triangles indicate both types.

**Figure 3:**
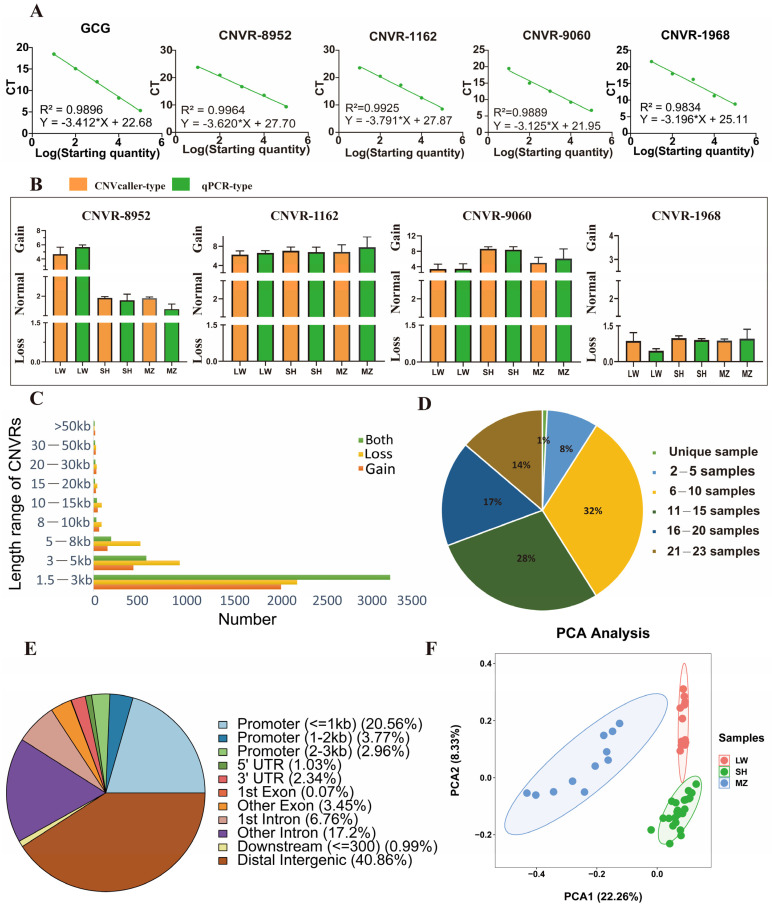
Descriptive statistics on CNVR type, CNVR length, genomic location, and frequency. (**A**) The standard curves of *GCG*, CNVR-8952, CNVR-1162, CNVR-9060, and CNVR-1968 were established according to the gradient concentration dilution method, respectively, and represent the amplification efficiency of the primers. (**B**) The CNV type between qPCR and CNVcaller assays in CNVR-8952, CNVR-1162, CNVR-9060, and CNVR-1968, respectively. (**C**) Length distribution of CNVRs, where the X-axis represents the number of CNVRs located in the length range, and the Y-axis represents the CNVRs’ length range. Green, yellow, and red represent both types, loss type, and gain type, respectively. (**D**) Frequency distribution of CNVRs. (**E**) Genomic location annotation of CNVRs. (**F**) PCA plot of LW, MZ, and SH pigs. Red, blue, and green colors represent the LW, MZ, and SH pigs, respectively. The variance proportions of PC1 and PC2 are 22.26% and 8.33%.

In total, 11,097 CNVRs were detected (Table S4), with 10,403 and 10,800 CNVRs identified in LW and MZ pigs, respectively (Figure 2). These CNVRs included 2885 gain, 4019 loss, and 4193 both types, spanning over 44 million base pairs and accounting for 1.85% of the porcine genome (Sscrofa 11.1) (Table 1). A standard curve was constructed using DNA gradient dilution to ensure efficient primer amplification (Figure 3A). Four types of CNVRs were validated using qPCR, which were consistent with those detected by CNVcaller (Figure 3B).

The lengths of the CNVRs were analyzed. The length of 1.5–3 kb was the most widely distributed, and was observed in 71.45%, 55.76%, and 77.82% of gain, loss, and both types, respectively (Figure 3C). Most CNVRs of both types showed lengths of 1.5–3 kb; thus, the shortest average length (total CNVR length/total CNVR number) was 3.3 kb (Table S5). Furthermore, 32% of CNVRs were found in 6–10 samples, and only 1% of CNVRs were found in unique samples (Figure 3D). The genomic positions of CNVRs were analyzed, and the largest proportion was positioned as distal intergenic (40.86%), followed by promoter (27.29%), intron (23.96%), exon (3.52%), and untranslated (UTR) regions (3.37%) (Figure 3E). A PCA plot was drawn for all samples based on the detected CNVRs, wherein LW, MZ, and SH pigs were divided into three groups. LW and SH pigs are closer in principal components because of their similar blood origin, and the variance proportions of PC1 and PC2 were 22.26% and 8.33% (Figure 3F).

### 3.2. Shared and Breed-Specific CNVRs

**Figure 4:**
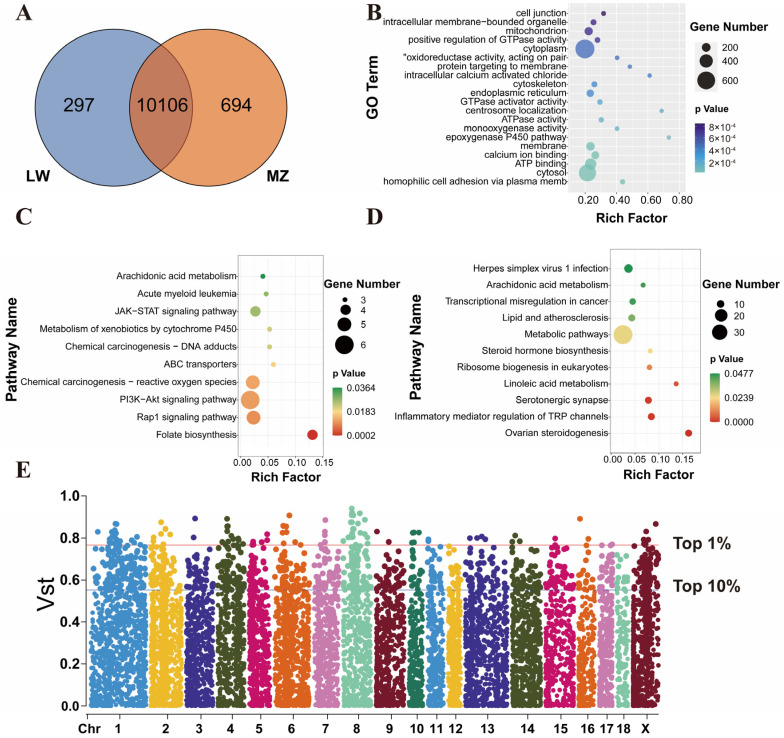
Analyses of shared and breed-specific CNVRs. (**A**) Venn diagram of the CNVRs in LW and MZ pigs, showing that 10,106 CNVRs were found to be shared between LW and MZ pigs, and 297 and 694 breed-specific CNVRs were present in LW and MZ pigs, respectively. (**B**) GO enrichment analysis was performed on annotated genes in shared CNVRs. (**C**,**D**) represent annotated genes in breed-specific CNVRs of LW and MZ pigs, respectively, on which KEGG enrichment analysis was performed. (**E**) Manhattan plot, representing the V_ST_ values of all CNVRs. CNVRs with the top 1% (*n* = 110) V_ST_ values were set as the preliminary differential CNVRs between LW and MZ pigs.

A Venn diagram was drawn to analyze the shared and breed-specific CNVRs between LW and MZ pigs (Figure 4A). In total, 10,106 CNVRs were found to be shared between LW and MZ pigs, and 297 and 694 breed-specific CNVRs were present in LW and MZ pigs, respectively. The genes overlapped by CNVRs were annotated, and 4606 genes were annotated in the shared CNVRs, including 2858 and 1748 known and novel genes, respectively (Table S6). GO enrichment analysis was performed to explore the functions of these genes. These genes were enriched in the cytoplasm, mitochondrion, and ATP binding, and may participate in the maintenance of basic physiological activities (Figure 4B). A total of 138 and 466 genes were annotated in the breed-specific CNVRs of LW and MZ pigs, respectively, including 94 and 44 known and novel genes in LW pigs, respectively, and 336 and 130 known and novel genes in MZ pigs, respectively. KEGG enrichment analysis was performed to explore the functions of breed-specific CNV genes. These genes of LW pigs were enriched in the JAK–STAT signaling pathway, PI3K-Akt signaling pathway, and Rap1 signaling pathway (Figure 4C). The breed-specific genes of MZ pigs were enriched in metabolic pathways, lipid and atherosclerosis, and ovarian steroidogenesis (Figure 4D). V_ST_ analysis was conducted to explore the differential CNVRs between LW and MZ pigs (Figure 4E), and CNVRs with the top 1% (*n* = 110) V_ST_ values were set as the preliminary differential CNVRs between LW and MZ pigs.

### 3.3. Multi-Omics Integration Analysis

**Figure 5:**
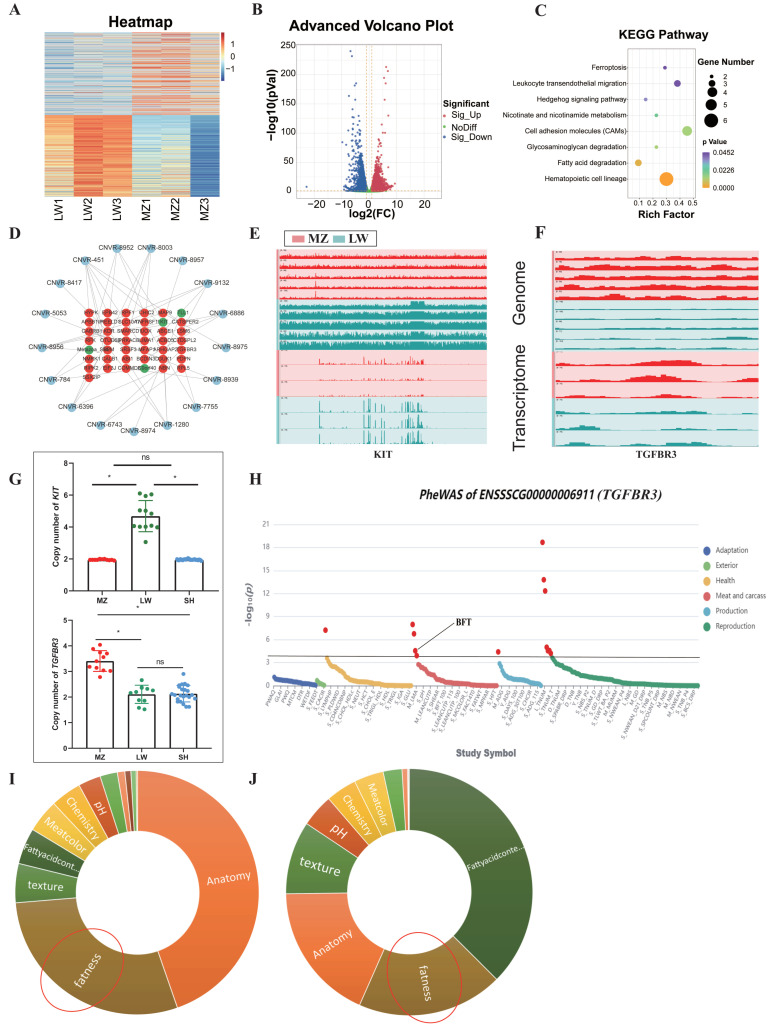
Multi-omics integrated analysis. (**A**) The heatmap represents a global expression of all genes in LW and MZ pigs. (**B**) The volcano plot represents the gene expression differences between LW and MZ pigs, where a total of 5317 genes were up-regulated and 3868 genes were down-regulated in MZ pigs relative to LW pigs. (**C**) KEGG enrichment analysis was performed on trans-eQTL effect genes. (**D**) Interactive network diagram between differential CNVRs and known genes with cis-eQTL effects. (**E**,**F**) represent the expression of *KIT* and *TGFBR3* at the genomic and transcriptomic level of MZ and LW pigs, respectively. (**G**) Copy number of *KIT* and *TGFBR3* in MZ, LW, and SH pigs, respectively (* *p* < 0.05, ns indicates no statistical difference). (**H**) The *TGFBR3* gene is significantly associated with BFT in pigs. (**I**) The eQTL effect CNVRs overlapped with the pigs’ QTLs, in which “fatness” (area circled in red) accounts for 28.99% of the “meat” trait QTLs. (**J**) In total pig QTL data, “fatness” (area circled in red) trait QTLs account for 19.13% of the “meat” trait QTLs.

The preliminarily differential CNVRs (*n* = 110) were combined with the integrative genomics viewer (IGV) to screen 22 CNVRs with larger structural differences between LW and MZ pigs (Figure S2). A heat map and a volcano plot were drawn to present the global genetic changes that were based on transcriptomic data of back fat from LW and MZ pigs (Figure 5A,B).

We performed trans-eQTL analysis to explore the relationship between the differential CNVRs and changes in gene expression over long distances. A total of 386 changes in gene expression were associated with differential CNVRs, including 382 upregulated genes and four downregulated genes (Table S7). KEGG enrichment showed that these genes participate in hematopoietic cell lineage, fatty acid degradation, and cell adhesion molecules (Figure 5C). It is notable that acyl-CoA oxidase 1 (ACOX1) and acyl-CoA synthetase long-chain family member 4 (ACSL4) involved in fatty acid metabolism were highly expressed in MZ pigs.

A cis-eQTL analysis was conducted to explore the relationship between the differential CNVRs and adjacent changes in gene expression. Nineteen of the 22 differential CNVRs influenced the expression of 67 genes, upregulating 59 genes and downregulating eight genes (Table S8). Among these, 43 are known genes, including 39 upregulated genes and four downregulated genes, which were affected by 17 CNVRs (Figure 5D), including *KIT* and *TGFBR3* gene down-regulation and up-regulation in MZ pigs, respectively. *KIT* controls melanin metabolism; therefore, *KIT* CNV polymorphism leads to completely white and black coat colors in LW and MZ pigs, respectively [40]. *TGFBR3* was reported to affect fat deposition and fatty acid metabolism [41]. Upon reevaluating the published correlations between *TGFBR3* and porcine phenotypes, we revealed a significant association between *TGFBR3* and BFT in pigs (Figure 5E) [42]. As a reference group, SH pigs exhibit a composite trait with lower BFT than MZ pigs and a black coat color. This is the same trend as the CNV of *KIT* and *TGFBR3* in SH pigs (Figure 5F). On the other hand, by integrating the expressions of *KIT* and *TGFBR3* genes at the genomic and transcriptomic levels, we noted similar trends for both. Thus, the CNVs of *KIT* and *TGFBR3* may have dose-related effects, increasing their transcriptomic expression (Figure 5G). The differential CNVRs with both trans-eQTL and cis-eQTL effects (*n* = 19) were selected and overlapped with the QTL data of the pigs. A total of 1414 QTLs were mapped in the CNVRs, including 51, 153, 1007, 141, and 62 QTLs in the “exterior”, “health”, “meat”, “production”, and “reproduction” trait categories, respectively (Table 2). The CNVRs with eQTL effect overlapped with the pigs’ QTLs, in which “fatness” accounts for 28.99% of the “meat” trait QTLs (Figure 5H). In the total pigs’ QTL data, “fatness” trait QTLs account for 19.13% of the “meat” trait QTLs (Figure 5I). 

### 3.4. Decreased Expression of TGFBR3 Inhibited Preadipocyte Proliferation and Differentiation

**Figure 6:**
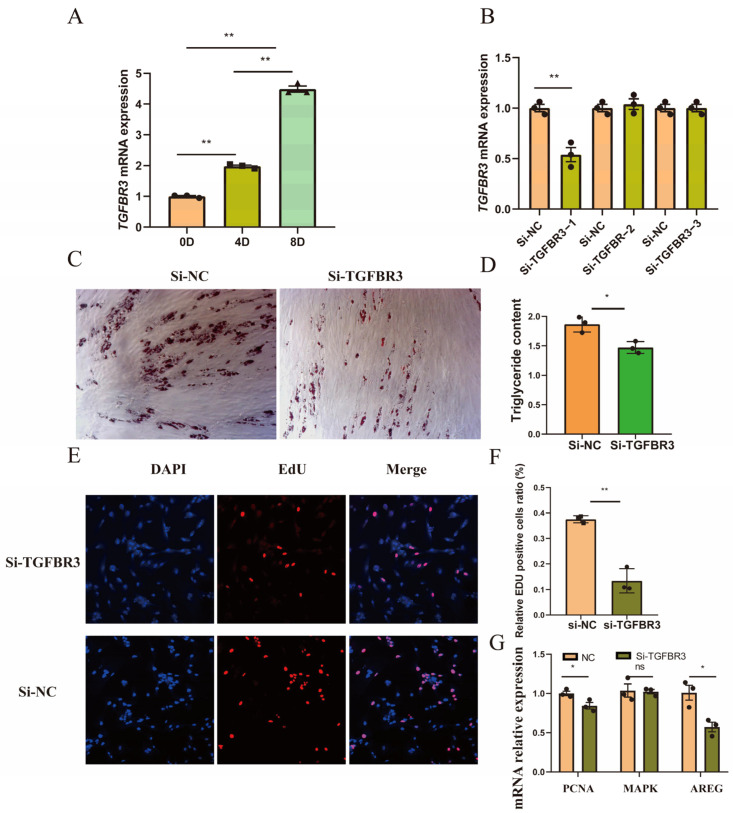
Decreased expression of *TGFBR3* inhibited preadipocyte proliferation and differentiation. (**A**) The expression levels of *TGFBR3* exhibit a gradual increase from 0 to 8 days post-induction (** *p* < 0.01). (**B**) si-TGFBR3−1 significantly reduced the expression level of *TGFBR3* (** *p* < 0.01). (**C**,**D**) Oil red O staining and triglyceride content assays confirmed that decreased expression of *TGFBR3* inhibited preadipocyte differentiation (* *p* < 0.05). (**E**,**F**) EdU staining on preadipocytes showed reduced expression of the *TGFBR3* gene significantly decreasing the cell proliferation rate of preadipocytes (** *p* < 0.01). (**G**) The cell proliferation-related genes *PCNA* and *AREG* were significantly reduced with transfection of si-TGFBR3−1 (* *p* < 0.05, ns indicates no statistical difference).

To investigate the role of *TGFBR3* in preadipocyte differentiation, porcine preadipocytes were induced using DIM, and the expression levels of *TGFBR3* were analyzed at 0 days, 4 days, and 8 days post-induction. The results revealed a progressive increase in the expression level of *TGFBR3* during the preadipocyte differentiation process (Figure 6A). Three siRNA sequences targeting *TGFBR3* were designed and transfected into porcine preadipocytes, as outlined in Table S2. Subsequent analysis conducted 2 days post-transfection indicated that si-TGFBR3−1 significantly decreased the expression level of *TGFBR3* (*p* < 0.05, Figure 6B). Furthermore, transfection of si-TGFBR3−1 into porcine preadipocytes, followed by oil red O staining and triglyceride content assays, confirmed that the decreased expression of *TGFBR3* inhibited preadipocyte differentiation (Figure 6C,D). Concurrently, EdU staining performed on preadipocytes transfected with si-TGFBR3−1 demonstrated that the reduced expression of *TGFBR3* notably decreased the proliferation rate of preadipocytes (Figure 6E,F). Moreover, the expression levels of cell proliferation-related genes, *PCNA* and *AREG*, were significantly reduced, as evidenced in Figure 6G.

## 4. Discussion

CNV is a structural variation in the genome that plays a crucial role in animal phenotypes and environmental adaptation [44]. Chinese indigenous pigs are genetically more diverse than European pigs [45]. In this study, we detected more CNVRs in MZ pigs (10,800) than in LW pigs (10,403), which is consistent with previous comparative studies between Meishan (8282) and Duroc (6700) pigs, as well as between Tongcheng (12,869) and LW (12,564) pigs [10,46]. A comprehensive study on six Chinese indigenous breeds and three European breeds revealed that 213 CNVRs were present in Chinese breeds, while only 60 CNVRs were found in European breeds [4].

In this study, the genomic positions of CNVRs were annotated. The majority (40.86%) of the CNVRs were located in the distal intergenic region, while only 3.52% were located in the exon region. This suggests that most CNVs may affect gene expression via noncoding regions. CNVs primarily influence gene expression through gene recombination and dose-related effects [47,48]. For example, the CNV located in the *MTHFSD* gene shows different copy number distributions across pig breeds and is closely correlated with litter size [49]. Additionally, a 38.7 kb CNVR in the *MSRB3* gene is significantly correlated with the expression of miR-584-5p, resulting in different ear sizes across pig breeds [50]. CNVs located on the *AHR* gene in pigs are strongly associated with litter size, and an increase in the copy number of the *AHR* gene leads to an increase in *AHR* gene expression. Studies have shown that abnormal *AHR* expression impairs the female reproductive system and affects reproductive performance in mice [14,51].

The process of DNA-to-protein conversion, as described by the central dogma, is complex and regulated by various factors. In particular, the transcription process from DNA to RNA is time- and tissue-dependent and requires precise transcription factors [52]. We investigated the effects of CNVs on the expression of candidate genes involved in fat deposition by analyzing genomic CNVRs and gene expression in back fat tissue through eQTL analysis.

Trans-eQTL analysis revealed that *ACOX1* and *ACSL4* were highly expressed and enriched in the fatty acid metabolic pathway in MZ pigs. *ACOX1* is the first rate-limiting enzyme in peroxisomal fatty acid β-oxidation and plays a crucial role in fatty acid metabolism and fat deposition [53]. *ACOX1* plays an important role in the intramuscular preadipocyte differentiation in chickens and cows [54]. In mouse models of human nonalcoholic fatty liver disease (NAFLD), *ACOX1* interacts with miR-222 and influences triglyceride formation in the cell [55]. This gene is of significant interest in human NAFLD lipid metabolism, as mutations in *ACOX1* lead to elevated plasma concentrations of long straight-chain fatty acids in humans, accompanied by developmental delay, cerebellar ataxia, and language impairment [56,57]. *ACOX1* has also been shown to significantly regulate lipid metabolism in model animals such as zebrafish, mice, and rats [55,58,59]. In bovine preadipocytes, C/EBPα and miR-25-3p target *ACOX1* to promote adipogenesis, which is significantly associated with bovine BFT and marbling [60]. An A/C polymorphism located in intron 9 of *ACOX1* significantly differs between Chinese indigenous fatty pigs and Western commercial pigs, and this SNP has been linked to fat deposition-related traits, including BFT and carcass fat percentage [61]. *ACSL4* is a member of the ACSL enzyme family, which preferentially catalyzes the metabolism of several polyunsaturated fatty acids, such as arachidonic acid [62]. *ACSL4* has been previously identified as a candidate gene that affects fat deposits in pigs, and subsequent integration of transcriptomic and proteomic data revealed the same conclusion [63,64]. It has been extensively reported to be involved in fat deposition and metabolism in pigs, potentially correlating with pig growth performance and meat quality [65]. Intramuscular fat (IMF) content is closely related to pork taste, and overexpression of *ACSL4* in intramuscular adipocytes increases the content of unsaturated fatty acids and promotes preadipocyte differentiation [66,67]. Although *ACSL4* polymorphisms have been significantly associated with BFT and ham weight in pigs, inconsistent findings have been reported across studies [68,69], which may be due to differences in pig breeds. However, *ACSL4* has been widely confirmed to be closely associated with fat deposition.

The cis-eQTL analysis showed that *TGFBR3* is highly expressed in MZ pigs. *TGFBR3* is a transmembrane proteoglycan involved in fat deposition. Overexpression of *TGFBR3* in human abdominal subcutaneous adipose stem cells promotes adipogenesis [70]. *TGFBR3* has also been implicated in lipid metabolism and IMF formation in Berkshire pigs [46]. CNVs of *TGFBR3* affect its gene expression through dose-related effects, as observed by integrating genomic and transcriptomic data. Similar mechanisms have been identified in human autism spectrum disorder and microbial resistance to drugs [71,72].

In the present study, the CNVR-related genes *ACOX1*, *ACSL4*, and *TGFBR3* were identified as key players in fatty acid metabolism. These genes were more highly expressed in the fatty MZ pigs compared to the lean LW pigs, suggesting that the expression of these CNV-related genes contributes to the BFT trait. We also employed a robust approach by combining genomic and transcriptomic data to identify candidate genes regulating BFT traits. 

## 5. Conclusions

In this study, we performed genome-wide CNV detection in LW and MZ pigs using next-generation sequencing data and explored the relationship between CNV-gene associations and BFT. We integrated genomic and transcriptomic data with eQTL analysis to investigate how CNVs influence gene expression. *TGFBR3* was identified as a candidate CNV gene for back fat deposition, with its expression level potentially linked to CNV-induced dose effects. Knockdown of *TGFBR3* in porcine preadipocytes resulted in the inhibition of proliferation and differentiation, suggesting that this CNVR-related gene may play a role in fat deposition in pigs. These findings enhance our understanding of back fat deposition mechanisms and provide new molecular genetic markers for the BFT trait in pigs.

## Figures and Tables

**Table 1:** CNVRs in LW and MZ pigs.

Breed	No. Sample	No. CNVR	No. Gains	No. Losses	No. Both	Length (bp)	Coverage, %
LW	12	10,403	3400	4454	2549	40,296,797	1.68
MZ	11	10,800	3827	5541	1432	41,886,800	1.75
Merge	23	11,097	2885	4019	4193	44,147,303	1.85

**Table 2:** Number of QTLs covered by CNVRs with cis_eqtl effect vs. the total number of QTLs in pigs [43].

Group	Total Number	Exterior	Health	Meat	Production	Reproduction	Fatness (Contained in Meat)	Fatness Proportion of Total QTLs (%)	Fatness Proportion of the Meat (%)
cis-eQTL_CNVR	1414	51	153	1007	141	62	292	20.65	28.99
Total QTLs	32,475	2492	6716	17,191	2911	3165	3289	10.13	19.13

## Data Availability

Data are contained within the article. The original contributions presented in the study are included in the article; further inquiries can be directed to the corresponding author.

## Supplementary Materials

The following supporting information can be downloaded at: https://www.mdpi.com/article/10.3390/ani14182657/s1. Table S1: Next-generation sequencing data and transcriptomic data of MZ and LW pigs; Table S2: Oligonucleotides used in this study; Table S3: Primers used in qPCR; Table S4: All CNVRs detected in the present study; Table S5: Length distribution and percentage of the CNVRs; Table S6: Genes annotated from the shared CNVRs of LW and MZ pigs; Table S7: Genes with trans-eQTL effects with differential CNVRs. Table S8: Genes with cis-eQTL effects with differential CNVRs; Figure S1: Linear analysis showed no significant correlation between sequencing depth and CNVR numbers; Figure S2: CNVRs (*n* = 22) with larger structural differences between LW and MZ pigs.
