# Supplementary material for: Genome-Wide Analysis Reveals Copy Number Variant Gene TGFBR3 Regulates Pig Back Fat Deposition
Source: Animals (Basel). 2024 Sep 12;14(18):2657. doi: 10.3390/ani14182657 (PMC11429474; doi:10.3390/ani14182657)
Supplement: Supplementary file 1 [file animals-14-02657-s001.zip › Figures S1 and S2.pdf]

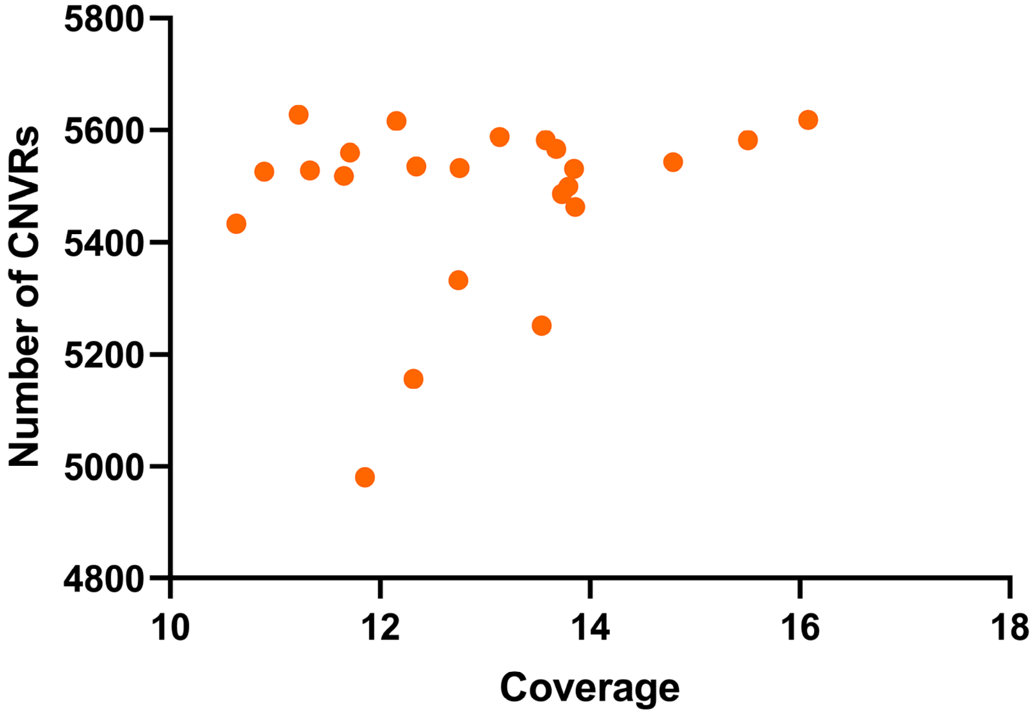

Figure S1. Linear analysis Showed no Significant correlation between Sequencing depth and CNVR numbers.

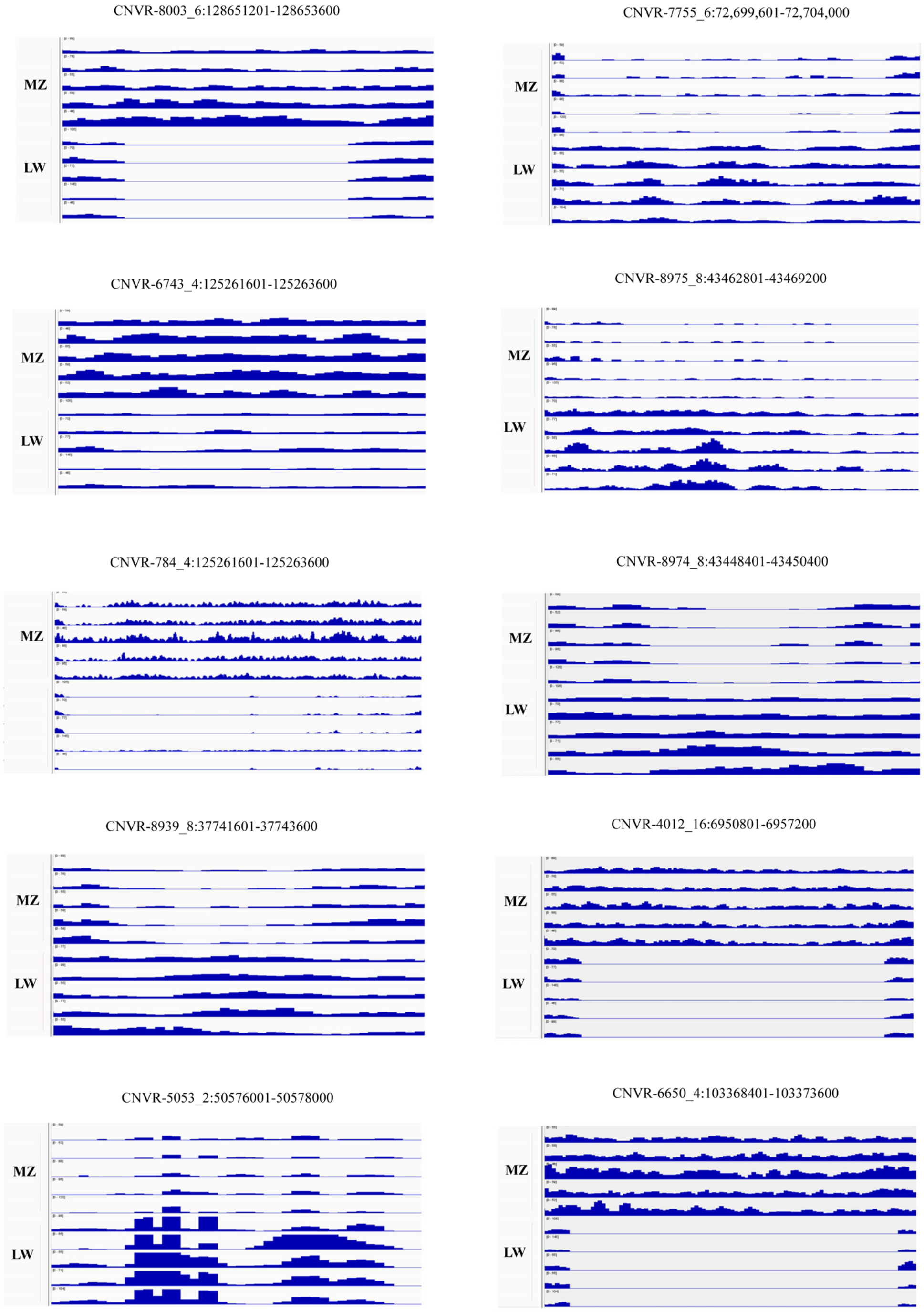
CNVR-8003_6:128651201-128653600
MZ
LW
CNVR-7755_6:72,699,601-72,704,000
MZ
LW
CNVR-6743_4:125261601-125263600
MZ
LW
CNVR-8975_8:43462801-43469200
MZ
LW
CNVR-784_4:125261601-125263600
MZ
CNVR-8974_8:43448401-43450400
MZ
LW
CNVR-8939_8:37741601-37743600
MZ
LW
CNVR-4012_16:6950801-6957200
MZ
LW
CNVR-5053_2:50576001-50578000
MZ
LW
CNVR-6650_4:103368401-103373600
MZ
LW

A

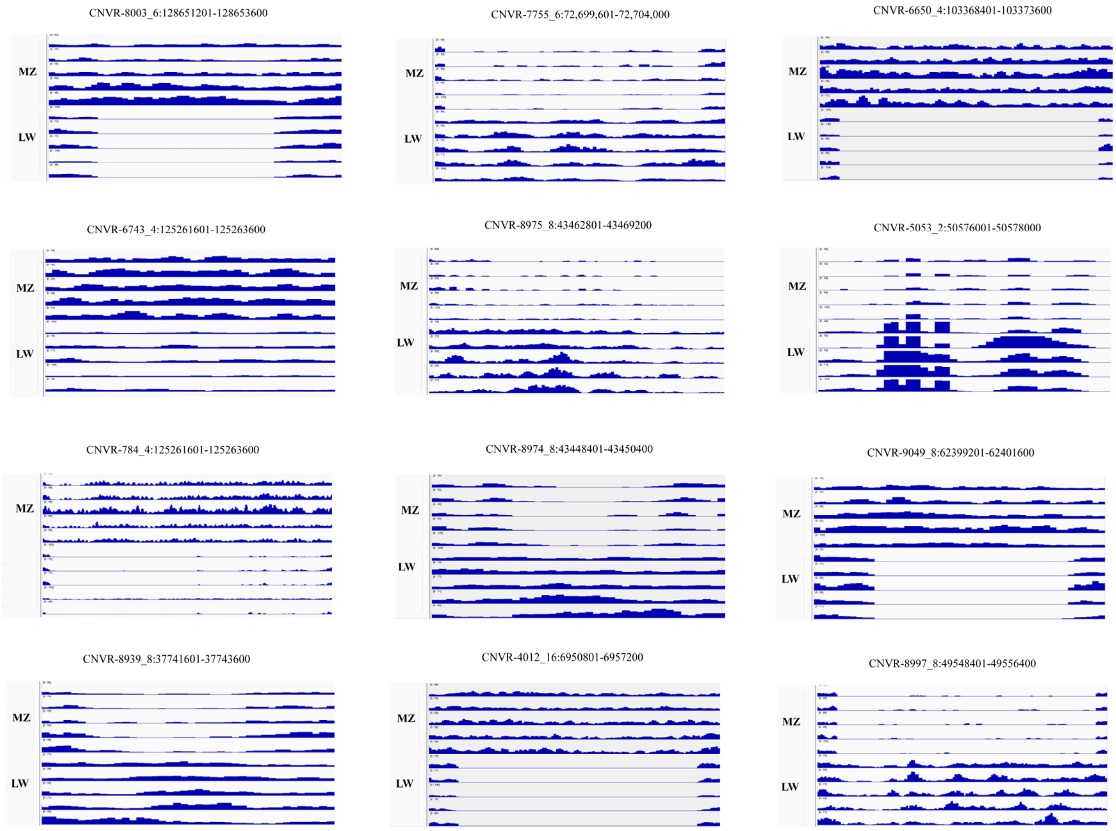

B

Figure S2. CNVRs (n = 22) with larger Structural differences between LWand MZ pigs.
